# An SVM-based system for predicting protein subnuclear localizations

**DOI:** 10.1186/1471-2105-6-291

**Published:** 2005-12-07

**Authors:** Zhengdeng Lei, Yang Dai

**Affiliations:** 1 Department of Bioengineering (MC063), University of Illinois at Chicago, 851 South Morgan Street, Chicago IL 60607, USA

## Abstract

**Background:**

The large gap between the number of protein sequences in databases and the number of functionally characterized proteins calls for the development of a fast computational tool for the prediction of subnuclear and subcellular localizations generally applicable to protein sequences. The information on localization may reveal the molecular function of novel proteins, in addition to providing insight on the biological pathways in which they function. The bulk of past work has been focused on protein subcellular localizations. Furthermore, no specific tool has been dedicated to prediction at the subnuclear level, despite its high importance. In order to design a suitable predictive system, the extraction of subtle sequence signals that can discriminate among proteins with different subnuclear localizations is the key.

**Results:**

New kernel functions used in a support vector machine (SVM) learning model are introduced for the measurement of sequence similarity. The *k*-peptide vectors are first mapped by a matrix of high-scored pairs of *k*-peptides which are measured by BLOSUM62 scores. The kernels, measuring the similarity for sequences, are then defined on the mapped vectors. By combining these new encoding methods, a multi-class classification system for the prediction of protein subnuclear localizations is established for the first time. The performance of the system is evaluated with a set of proteins collected in the Nuclear Protein Database (NPD). The overall accuracy of prediction for 6 localizations is about 50% (vs. random prediction 16.7%) for single localization proteins in the leave-one-out cross-validation; and 65% for an independent set of multi-localization proteins. This integrated system can be accessed at .

**Conclusion:**

The integrated system benefits from the combination of predictions from several SVMs based on selected encoding methods. Finally, the predictive power of the system is expected to improve as more proteins with known subnuclear localizations become available.

## Background

The cell nucleus is a highly complex organelle that organizes the comprehensive assembly of our genes and their corresponding regulatory factors. Accordingly, the cell nucleus reflects the intricate regulation of various biological activities. Although protein complexes disperse throughout the entire organelle, it is known that many nuclear proteins participating in related pathways tend to concentrate into specific areas [1,2]. For example, the rDNA processing and ribosome biogenesis often occur within the nucleolus and the proteins responsible for pre-splicing appear to concentrate into multiple nuclear speckles, even while they are migrating in the nucleus. The confinement of biomolecules within specific compartments is crucial for the formation and function of the cell nucleus; in contrast, the mis-localization of proteins can lead to both human genetic disease and cancer [3].

Accordingly, information on protein subnuclear localization is essential for a full understanding of genomic regulation and function. Advances in experimental technology have enabled the large-scale identification of nuclear proteins. However, at the same time, the sequencing of both the human and mouse genomes has generated an enormous inventory of primary sequences with unknown functions. A faster and cheaper bioinformatics tool is required for the annotation of these exponentially accumulating sequences. A computational prediction of protein subnuclear compartments from primary protein sequences can provide important clues to the function of novel proteins.

A host of systems for the prediction of protein subcellular localizations has emerged over the last two decades [4-23]. This list includes several web-based predictors that have a broad coverage of subcellular localizations at the genomic level, such as PSORT [4], SubLoc [7], Proteome Analyst [15], CELLO [16], PSORTb v.2.0 [17], and LOCtree [21]. The development led to the ability to predict the particular subcellular compartment, in which a given protein resides within a cell, with a steadily increasing accuracy. The predictions for eukaryotic organisms, however, have certain limitations. They can provide information on whether a protein localizes in the nuclear compartment, but they can not discriminate among the sub-compartments in which it functions.

The prediction of protein localization at the subnuclear level is challenging compared with that at the subcellular level. Three facts contribute to the difficulty: (1) proteins within the cell nucleus face no apparent physical barrier like a membrane [24]; (2) the nucleus is far more compact and complicated in comparison with other compartments in a cell [25]; and (3) protein complexes within the cell nucleus are not static [1,24,25]. Recent developments in live-cell imaging have revealed that nuclear processes may rely on a constant flow of molecules between dynamic compartments created by relatively immobile binding or assembly sites. As proteins diffuse through the nuclear space, they appear to alter their compartments during different phases of the cell cycle or accompanying differentiation [3]. For instance, some nucleolar proteins are continually exchanging between the nucleoplasm and the nucleolus. Proteomic studies have also highlighted the dynamic nature of the nucleolar proteome [3].

Employing the database Nuclear Protein Database (NPD) developed by Dellaire, Farrall and Bickmore [26], Bickmore and Sutherland [27] recently addressed the characteristics of the primary sequences of nuclear proteins, such as the molecular weight, isoelectric point, and amino acid composition for proteins in different subnuclear compartments. They also found that motifs and domains are often shared by proteins co-localized within the same subnuclear compartment. Furthermore, certain generally abundant motifs/domains are lacking from the proteins concentrated in some specific areas of the nucleus. Based on these findings, it should be possible to combine totality of this information in a manner that will enhance the prediction of compartmental-specific nuclear localizations of the protein constituents listed in genome databases.

Encouraged by our previous success in the design of a metric for the biological similarity of protein sequences [22,23], a prediction system is developed based on support vector machines (SVMs), one of the most advanced machine learning methods [28,29]. The principal feature of our mode of analysis is the introduction of new kernel functions which are effective in capturing the subtle difference between sequences originated from two distinct nuclear compartments.

## Results and Discussion

Normally, conventional *k*-peptide encoding vectors (*k *= 1, 2, 3) are used for the description of a protein sequence. Successful applications include (1) the protein fold recognition [30,31], and (2) the prediction of subcellular localization [5,7,16]. The basic concept of the new kernels proposed in our previous work [22,23] is the measurement of biological similarity for *k*-peptides, having either none or a few shared residues, with the incorporation of evolutionary information. Our finding indicates that the mapping of conventional *k*-peptide encoding vectors by a matrix formed with high-scored pairs of *k*-peptides can facilitate the construction of a suitable metric. The score of a pair of *k*-peptides is calculated by the BLOSUM scores of residues and, therefore, the evolutionary information of the residues is embedded into the sequence description. A related concept that links two *k*-peptides with a small number of mutated residues has been presented by Leslie *et al*. [32] for protein homology detection.

This study presents the performance of conventional *k*-peptide encoding methods and the new proposed kernels for the prediction of protein subnuclear compartments. Furthermore, with the use of the jury voting scheme developed in [31], an integrated system was built by combining binary prediction outcomes obtained from different sequence encoding schemes. The results demonstrate that the integrated system enhances the overall performance of the system.

The dataset used in this study was extracted from the Nuclear Protein Database (NPD) [26] using a Perl script. The NPD is a curated database that stores information on more than 1000 vertebrate proteins, chiefly from human and mouse, which are reported in the literature to be localized in the cell nucleus. Since certain proteins associate with more than one compartment, a test dataset consisting of proteins with multiple localizations was first extracted out. These proteins have the same SwissProt ID or Entrez Protein ID though localized in different compartments. This preparative procedure resulted in 92 proteins that are localized within the six compartments described below. The majority is localized in 2 compartments and the remaining portion is localized in 3 or 4 compartments.

After excluding the multi-localization proteins, a non-redundant dataset was further constructed by PROSET [33] to ensure low sequence identity (<50%). In order to have sufficient number of proteins for training and testing, only six localizations were selected for evaluation. These are PML BODY (38), Nuclear Lamina (55), Nuclear Splicing Speckles (56), Chromatin (61), Nucleoplasm (75), and Nucleolus (219). Each of these proteins has a single localization and the total number is 504.

It should be noted that the multi-localization proteins are not included in the set of 504 single-localization proteins for the leave-one-out cross-validation (LOOCV). Therefore, the multi-localization dataset is essentially an independent testing set. The summary of the datasets is presented in Table 1.

**Table 1:** The summary of the nuclear proteins

Label	Compartment	Number of sequences
1	PML BODY	38
2	Nuclear Lamina	55
3	Nuclear Splicing Speckles	56
4	Chromatin	61
5	Nucleoplasm	75
6	Nucleolus	219
-	Multiple Localizations	92

AA – amino acid composition encoding method;

DI – di-peptide encoding method;

TRI – tri-peptide encoding method;

D_1_X_1 _– amino acid composition encoding vector transformed with D_1_;

D_2_X_2 _– di-peptide encoding vector transformed with D_2_;

D_3_X_3 _– tri-peptide encoding vector transformed with D_3_.

The evaluations of the predictive power of the methods were performed on the datasets. Since there are 6 localizations in the dataset, the one-versus-one multi-class classification system led to 6*(6-1)/2 = 15 SVM models for one single encoding method (see Methods for details). Three encoding techniques corresponding to the conventional k-peptide composition and three encoding methods based on the new kernels were used for k = 1,2,3. SVMLight [34] was used as the SVM solver.

The overall accuracy for the multi-class classification proposed by Rost and Sander [35] was used for the evaluation of our system. Suppose there are *m *= *m*_1 _+ *m*_2 _+ ... + *m*_*N *_test proteins, where *m*_*i *_is the number of proteins belonging to class *i*(*i *= 1,...,*N*). Suppose further that out of the proteins considered, *p*_*i *_proteins are correctly predicted to belong to class *i*. Then *p *= *p*_1 _+ *p*_2 _+ ... + *p*_*N *_is the total number of correctly predicted proteins. The accuracy for class *i *is

acci = pimi,
 MathType@MTEF@5@5@+=feaafiart1ev1aaatCvAUfKttLearuWrP9MDH5MBPbIqV92AaeXatLxBI9gBaebbnrfifHhDYfgasaacH8akY=wiFfYdH8Gipec8Eeeu0xXdbba9frFj0=OqFfea0dXdd9vqai=hGuQ8kuc9pgc9s8qqaq=dirpe0xb9q8qiLsFr0=vr0=vr0dc8meaabaqaciGacaGaaeqabaqabeGadaaakeaacqWGHbqycqWGJbWycqWGJbWydaWgaaWcbaGaemyAaKgabeaakiabbccaGiabg2da9iabbccaGmaalaaabaGaemiCaa3aaSbaaSqaaiabdMgaPbqabaaakeaacqWGTbqBdaWgaaWcbaGaemyAaKgabeaaaaGccqqGSaalaaa@3B99@

and the overall accuracy, denoted by Q_acc_, is defined as

Qacc=∑i=1Nacci×mim=∑i=1Npim=pm.
 MathType@MTEF@5@5@+=feaafiart1ev1aaatCvAUfKttLearuWrP9MDH5MBPbIqV92AaeXatLxBI9gBaebbnrfifHhDYfgasaacH8akY=wiFfYdH8Gipec8Eeeu0xXdbba9frFj0=OqFfea0dXdd9vqai=hGuQ8kuc9pgc9s8qqaq=dirpe0xb9q8qiLsFr0=vr0=vr0dc8meaabaqaciGacaGaaeqabaqabeGadaaakeaacqWGrbqudaWgaaWcbaGaemyyaeMaem4yamMaem4yamgabeaakiabg2da9maaqahabaGaemyyaeMaem4yamMaem4yam2aaSbaaSqaaiabdMgaPbqabaGccqGHxdaTdaWcaaqaaiabd2gaTnaaBaaaleaacqWGPbqAaeqaaaGcbaGaemyBa0gaaaWcbaGaemyAaKMaeyypa0JaeGymaedabaGaemOta4eaniabggHiLdGccqGH9aqpdaaeWbqaamaalaaabaGaemiCaa3aaSbaaSqaaiabdMgaPbqabaaakeaacqWGTbqBaaGaeyypa0ZaaSaaaeaacqWGWbaCaeaacqWGTbqBaaaaleaacqWGPbqAcqGH9aqpcqaIXaqmaeaacqWGobGta0GaeyyeIuoakiabc6caUaaa@56B3@

Note that *acc*_*i *_and *Q*_*acc *_are respectively corresponding to the definitions of Qi%obs
 MathType@MTEF@5@5@+=feaafiart1ev1aaatCvAUfKttLearuWrP9MDH5MBPbIqV92AaeXatLxBI9gBaebbnrfifHhDYfgasaacH8akY=wiFfYdH8Gipec8Eeeu0xXdbba9frFj0=OqFfea0dXdd9vqai=hGuQ8kuc9pgc9s8qqaq=dirpe0xb9q8qiLsFr0=vr0=vr0dc8meaabaqaciGacaGaaeqabaqabeGadaaakeaacqWGrbqudaqhaaWcbaGaemyAaKgabaGaeiyjauIaem4Ba8MaemOyaiMaem4Camhaaaaa@3456@ and *Q*_*total *_in Rost and Sander [35]. Since the numbers of proteins for various localizations are unbalanced, the Matthew's correlation coefficient (MCC) was also employed for the optimization of parameters and evaluation of performance [36]:

MCCi=pisi−uioi(pi+ui)(pi+oi)(si+ui)(si+oi),
 MathType@MTEF@5@5@+=feaafiart1ev1aaatCvAUfKttLearuWrP9MDH5MBPbIqV92AaeXatLxBI9gBaebbnrfifHhDYfgasaacH8akY=wiFfYdH8Gipec8Eeeu0xXdbba9frFj0=OqFfea0dXdd9vqai=hGuQ8kuc9pgc9s8qqaq=dirpe0xb9q8qiLsFr0=vr0=vr0dc8meaabaqaciGacaGaaeqabaqabeGadaaakeaacqWGnbqtcqWGdbWqcqWGdbWqdaWgaaWcbaGaemyAaKgabeaakiabg2da9maalaaabaGaemiCaa3aaSbaaSqaaiabdMgaPbqabaGccqWGZbWCdaWgaaWcbaGaemyAaKgabeaakiabgkHiTiabdwha1naaBaaaleaacqWGPbqAaeqaaOGaem4Ba82aaSbaaSqaaiabdMgaPbqabaaakeaadaGcaaqaaiabcIcaOiabdchaWnaaBaaaleaacqWGPbqAaeqaaOGaey4kaSIaemyDau3aaSbaaSqaaiabdMgaPbqabaGccqGGPaqkcqGGOaakcqWGWbaCdaWgaaWcbaGaemyAaKgabeaakiabgUcaRiabd+gaVnaaBaaaleaacqWGPbqAaeqaaOGaeiykaKIaeiikaGIaem4Cam3aaSbaaSqaaiabdMgaPbqabaGccqGHRaWkcqWG1bqDdaWgaaWcbaGaemyAaKgabeaakiabcMcaPiabcIcaOiabdohaZnaaBaaaleaacqWGPbqAaeqaaOGaey4kaSIaem4Ba82aaSbaaSqaaiabdMgaPbqabaGccqGGPaqkaSqabaaaaOGaeiilaWcaaa@62BA@

where *p*_*i *_is the number of correctly predicted proteins of the location *i*, *s*_*i *_is the number of correctly predicted proteins not in the location *i*, *u*_*i *_is the number of under-predicted proteins, and *o*_*i *_the number of over-predicted proteins.

In order to evaluate the performance of the system for multi-localization proteins, the criterion proposed in Gardy *et al*. was used [17]. More specifically, for a protein with multi-localization, if the system validly predicts one of the locations, then the entire prediction is considered correct. It should be noted that this criterion overestimates the performance. Since our method can only predict one localization for a given protein, other evaluation methods for multi-localization proteins such as the one proposed by Chou and Cai [14,18] can not be applied.

The performances for each encoding method and the combined encoding methods are shown in Table 2 and Table 3, respectively. The results for the single-localization proteins were obtained from the LOOCV procedure; and the results for the multi-localization proteins were obtained from the final prediction system. Overall, the single encoding methods gave an accuracy of prediction *Q*_*acc *_that ranged from 47.8% to 51.4% for single-localization proteins and from 57.6% to 64.1% for multi-localization proteins. The corresponding average MCCs ranged from 0.203 to 0.276 for single-localization proteins and from 0.182 to 0.401 for multi-localization proteins. The combination of the new encoding methods D_1_X_1_, D_2_X_2_, and D_3_X_3 _with the use of jury voting yielded an improved performance for MCC. For example, the average MCC was elevated from 0.266–0.276 to 0.284 for single-localization proteins and from 0.362–0.401 to 0.420 for multi-localization proteins. The change in *Q*_*acc *_was not uniform: it decreased from the highest value 51.4% to 50.0% for single-localization protein and increased from 64.1% to 65.2% for multi-localization proteins. The combination of the conventional *k*-peptide compositions AA, DI, and TRI did not demonstrate significant improvement. Further optimization of the parameter for the determination of sparsity of matrix *D*_3 _is likely to enhance the performance of the prediction system.

**Table 2:** Results for each individual encoding method

Method	AA	DI	TRI	D_1_X_1_	D_2_X_2_	D_3_X_3_
Compartment	Accuracy % [MCC]					

PML BODY	26.3 [0.144]	13.2 [0.091]	0.0 [-0.045]	31.6 [0.183]	29.0 [0.139]	10.5 [0.066]
Nuclear Lamina	40.0 [0.363]	27.3 [0.256]	40.0 [0.228]	45.5 [0.340]	41.8 [0.279]	36.4 [0.331]
Nuclear Splicing Speckles	30.4 [0.326]	32.1 [0.358]	30.4 [0.365]	33.9 [0.321]	33.9 [0.316]	33.9 [0.391]
Chromatin	14.8 [0.174]	11.5 [0.106]	13.1 [0.191]	19.8 [0.215]	21.3 [0.248]	21.3 [0.271]
Nucleoplasm	25.3 [0.189]	26.7 [0.207]	12.0 [0.123]	20.0 [0.182]	22.7 [0.246]	28.0 [0.229]
Nucleolus	78.1 [0.374]	83.1 [0.357]	85.8 [0.357]	73.5 [0.357]	72.2 [0.364]	83.1 [0.367]
Single-localization Overall Accuracy and MCC	**49.2 [0.262]**	**49.0 [0.229]**	**48.4 [0.203]**	**48.4 [0.266]**	**47.8 [0.265]**	**51.4 [0.276]**
Multi-localization Overall Accuracy and MCC	**64.1 [0.365]**	**57.6 [0.343]**	**58.7 [0.182]**	**60.9 [0.401]**	**57.6 [0.362]**	**64.1 [0.362]**

AA – amino acid composition encoding method;

DI – di-peptide encoding method;

TRI – tri-peptide encoding method;

D_1_X_1 _– amino acid composition encoding vector transformed with D_1_;

D_2_X_2 _– di-peptide encoding vector transformed with D_2_;

D_3_X_3 _– tri-peptide encoding vector transformed with D_3_.

**Table 3:** Results using combined methods

Methods	Combination of AA, DI, TRI	Combination of D_1_X_1_, D_2_X_2_, and D_3_X_3_
Compartment	Accuracy % [MCC]	

PML BODY	13.2 [0.073]	29.0 [0.172]
Nuclear Lamina	30.9 [0.275]	43.6 [0.338]
Nuclear Splicing Speckles	32.1 [0.410]	35.7 [0.363]
Chromatin	9.8 [0.170]	19.7 [0.260]
Nucleoplasm	20.0 [0.182]	22.7 [0.206]
Nucleolus	88.1 [0.374]	76.7 [0.367]
Single-localization Overall Accuracy and MCC	**50.4 [0.247]**	**50.0 [0.284]**
Multi-localization Overall Accuracy and MCC	**62.0 [0.362]**	**65.2 [0.420]**

AA – amino acid composition encoding method;

DI – di-peptide encoding method;

TRI – tri-peptide encoding method;

D_1_X_1 _– amino acid composition encoding vector transformed with D_1_;

D_2_X_2 _– di-peptide encoding vector transformed with D_2_;

D_3_X_3 _– tri-peptide encoding vector transformed with D_3_.

The final models for the prediction system are the combination of the new encoding methods D_1_X_1_, D_2_X_2_, and D_3_X_3_, since adding any conventional *k*-peptide encoding method does not improve the performance of the system. The predictions for all the 92 multi-localization testing proteins are detailed in Table S1 in the supplementary file [see Additional file 1].

## Conclusion

An SVM-based multi-class classification system has been developed for the prediction of protein subnuclear localizations. This is the first system designed specifically for this task. This system, which integrates predictions from three new encoding methods, achieves encouraging levels of accuracy for six specific subnuclear localizations. However, compared to the prediction of protein localizations at the subcellular level, the corresponding prediction at the subnuclear level is far more challenging. This difficulty arises mainly from the biological fact that each compartment within the cell nucleus contains no apparent physical barrier like a membrane. Furthermore, the nucleus is a considerably more compact and complex organelle in comparison to other organelles in the cell. Finally, the dynamic nature of the nucleolar proteome adds an additional level of complexity to the task of prediction.

## Methods

### Kernels based on high-scored pairs of *k*-peptides

Recently, Lei and Dai proposed new kernels based on high-scored pairs of *k*-peptides for protein sequence encoding [22,23] for the SVMs. Superior performance of the SVMs with these new kernels was demonstrated through application to the prediction of protein subcellular localization. The kernels proposed in [22,23] can be described as follows.

A matrix *D*_*k *_of high scored *k*-peptide pairs is defined with a prescribed threshold. Each entry is associated with the BLOSUM score of some pair of *k*-peptides. The matrix is of dimension 21^*k *^× 21^*k*^, where 21 is the number of amino acid symbols (normal 20 amino acids plus the special symbol ''X''). The thresholds are set to zeroes for *k *= 1, 2. Therefore, matrix *D*_1 _is the same as the BLOSUM matrix, except that the entries with negative values are replaced by zeroes; the entries of matrix *D*_2 _are the BLOSUM pair scores of two di-peptides with all negative values being replaced by zeroes. Since the size of *D*_3 _is very large and the majority of all possible pairs is associated with lower scores, the elimination of those pairs can reduce noise that may confuse the prediction. Therefore, a careful thresholding is necessary to ensure the sparsity of the matrix *D*_3_. In this work, the threshold is set to 8 for *k *= 3. For example, the score is 12 for an AAA-AAA pair, 11 for an AAY-ACY pair, and 0 for a TVW-TVR pair since TVW-TVR BLOSUM62 pair-score is 6, which is smaller than the threshold value 8. Given the dimensional scaling, when *k *> 3, such a coding scheme is less attractive from a computational point of view.

For a pair of *k*-peptide composition vectors *x*_*ki*_, *x*_*kj*_, the new kernels are defined as

*K *(*x*_*ki*_, *x*_*kj*_) = exp(-*γ *|| *D*_*k*_*x*_*ki *_- *D*_*k*_*x*_*kj *_||^2^), k = 1, 2, 3, ....

It can be considered as a Gaussian kernel for a pair of vectors *D*_*k*_*x*_*ki *_and *D*_*k*_*x*_*kj*_. These kernels define the sequence similarity for the mapped vectors *D*_*k*_*x*_*ki *_and *D*_*k*_*x*_*kj*_, not directly for the *k*-peptide composition vectors *x*_*ki *_and *x*_*kj*_. In this study, the kernel type used for the conventional *k*-peptide composition encoding methods is the radial basis kernel: exp(-*γ *|| *x*_*ki *_- *x*_*kj *_||^2^)

In the following, the concept described above is illustrated and the comparison with the conventional *k*-peptide encoding method is provided. Consider two short amino acid sequences AAACY and AACCY. Using the input format of the SVMLight [34], the conventional tri-peptide encoding method generates two coding vectors:

*x*_31_: 1:0.33 **2:0.33 **42:0.33

*x*_32_: **2:0.33 **23:0.33 483:0.33

where the numbers appearing in the vectors are in the format of "index: score". It is obvious that the two sequences share the tri-peptide "AAC", and the corresponding vector index is 2. On the other hand, using BLOSUM62, the transformed vectors *D*_3_*x*_31 _for *x*_31 _and *D*_3_*x*_32 _for *x*_32 _are calculated as follows:

Example of encoding AAACY to *D*_3_*x*_31_:

ACY0000......11......0AAC8170000AAA1280000AAAAACAADAAE......AAY......YYY↓↓↓↓↓↓6.678.3300......3.670
 MathType@MTEF@5@5@+=feaafiart1ev1aaatCvAUfKttLearuWrP9MDH5MBPbIqV92AaeXatLxBI9gBaebbnrfifHhDYfgasaacH8akY=wiFfYdH8Gipec8Eeeu0xXdbba9frFj0=OqFfea0dXdd9vqai=hGuQ8kuc9pgc9s8qqaq=dirpe0xb9q8qiLsFr0=vr0=vr0dc8meaabaqaciGacaGaaeqabaqabeGadaaakeaafaqabeGbjaaaaaaabaGaeeyqaeKaee4qamKaeeywaKfabaGaeGimaadabaGaeGimaadabaGaeGimaadabaGaeGimaadabaGaeiOla4IaeiOla4IaeiOla4IaeiOla4IaeiOla4IaeiOla4cabaGaeGymaeJaeGymaedabaGaeiOla4IaeiOla4IaeiOla4IaeiOla4IaeiOla4IaeiOla4cabaGaeGimaadabaGaeeyqaeKaeeyqaeKaee4qameabaGaeGioaGdabaGaeGymaeJaeG4naCdabaGaeGimaadabaGaeGimaadabaaabaGaeGimaadabaaabaGaeGimaadabaGaeeyqaeKaeeyqaeKaeeyqaeeabaGaeGymaeJaeGOmaidabaGaeGioaGdabaGaeGimaadabaGaeGimaadabaaabaGaeGimaadabaaabaGaeGimaadabaaabaGaeeyqaeKaeeyqaeKaeeyqaeeabaGaeeyqaeKaeeyqaeKaee4qameabaGaeeyqaeKaeeyqaeKaeeiraqeabaGaeeyqaeKaeeyqaeKaeeyraueabaGaeiOla4IaeiOla4IaeiOla4IaeiOla4IaeiOla4IaeiOla4cabaGaeeyqaeKaeeyqaeKaeeywaKfabaGaeiOla4IaeiOla4IaeiOla4IaeiOla4IaeiOla4IaeiOla4cabaGaeeywaKLaeeywaKLaeeywaKfabaaabaGaey4KH8kabaGaey4KH8kabaGaey4KH8kabaGaey4KH8kabaaabaGaey4KH8kabaaabaGaey4KH8kabaaabaGaeGOnayJaeiOla4IaeGOnayJaeG4naCdabaGaeGioaGJaeiOla4IaeG4mamJaeG4mamdabaGaeGimaadabaGaeGimaadabaGaeiOla4IaeiOla4IaeiOla4IaeiOla4IaeiOla4IaeiOla4cabaGaeG4mamJaeiOla4IaeGOnayJaeG4naCdabaaabaGaeGimaadaaaaa@9240@

*D*_3_*x*_31_: **1:6.67 2:8.33 **6:2.67 16: 3.00 17:2.67 18:2.67 21: 3.67 **22:6.33 23:8.00 24:3.33 25:3.67 26:5.33 27:3.33 28:5.00 29:4.00 30:3.67 **...

*D*_3_*x*_32_: **1:2.67 2:10.00 22:4.33 23:11.67 24:3.33 25:3.00 26:7.67 27:3.33 28:7.00 **...

From the list it is seen that the transformed vectors share more common indices, such as 1, 2, 22–28 etc. Therefore, the similarity between the two sequences is more likely to be captured by the new methods even they do not share explicitly those tri-peptides. The mismatch string kernels proposed in Leslie *et al*. [32] also consider the similarity between mismatch *k*-peptides. For example, compared with the conventional tri-peptide encoding, the two sequences share several more common tri-peptides, such as AAA and AAC, AAC and ACC, ACY and CCY, if one mismatch is allowed in two peptides. Therefore, our method is related to the mismatch string kernel but it is different.

### Multi-class classification system

The efficient extension of SVMs to the handling of multiple classes has been achieved for applications to protein fold prediction [30] and the prediction of subcellular localization [7,16]. The one-versus-one [37] framework was used here for the assembly of the multi-class classifier from binary classifiers. For a classification problem of *N *class, it trains every pair-wise binary classifier. This gives a total of 1/2 * *N *(*N *- 1) classifiers. The prediction of the label of a testing protein follows the jury voting; specifically, sum the predictions for each classifier and take the label with the highest votes. When ties arise, the class label is assigned to the class with the maximum value of the sum of the function margins. This jury voting scheme is very flexible for the assembly of the predictions obtained from various SVM models. It can integrate not only the outcome from binary predictors with one encoding scheme, but also those obtained from alternative encoding methods. Accordingly, the class label of the testing protein is assigned to the class with the maximum votes.

### Cross-validation and final prediction system

The generalization performance of an SVM is controlled by the following parameters:

(1) C: the trade-off between the training error and class separation;

(2) *γ*: the parameter in the radial basis functions exp(-*γ *|| *x*_*i *_- *x*_*j *_||^2^) or exp(-*γ *|| *D*_*k*_*x*_*ki *_- *D*_*k*_*x*_*kj *_||^2^);

(3) J: the biased penalty for errors from positive and negative training points.

The leave-one-out cross-validation (LOOCV) was employed for the evaluation. The LOOCV is also referred as jackknife test, which is considered to be more rigorous and reliable compared with other testing techniques. A justification of the rigorousness and reliability of the LOOCV can be found, e.g., in Chou and Zhang [38]. Assume that there are overall *m *proteins. Each protein was in turn considered as a testing protein and the parameters associated with the SVM model were optimized based on a 5-fold cross-validation by using the remaining *m *- 1 proteins. The criterion of the optimization is the sum of the Matthew's correlation coefficients over all classes [36]. The final LOOCV classifiers were determined by using the optimized parameters to train the set of the *m *- 1 proteins. The search ranges corresponding to the parameters in the 5-fold cross validation optimization are the following:

(1) *C*: 2^-2^, 2^-1^, 1, ..., 2^9^, 2^10^;

(2) *γ*: 2^-15^, 2^-14^, 2^-13^, ..., 2^14^, 2^15^;

(3) *J*: 1, 2, 3, ..., 8, 9.

The labels of the training sets were arranged in a way that the size of the negative set is always larger than that of the positive set in our experiment. Here, the penalty term C∑iξi
 MathType@MTEF@5@5@+=feaafiart1ev1aaatCvAUfKttLearuWrP9MDH5MBPbIqV92AaeXatLxBI9gBaebbnrfifHhDYfgasaacH8akY=wiFfYdH8Gipec8Eeeu0xXdbba9frFj0=OqFfea0dXdd9vqai=hGuQ8kuc9pgc9s8qqaq=dirpe0xb9q8qiLsFr0=vr0=vr0dc8meaabaqaciGacaGaaeqabaqabeGadaaakeaacqWGdbWqdaaeqbqaaiabe67a4naaBaaaleaacqWGPbqAaeqaaaqaaiabdMgaPbqab0GaeyyeIuoaaaa@347A@ in the SVM is split into two terms: C∑iξi=C∑{i:yi=1}ξi+CJ∑{i:y=−1}ξi
 MathType@MTEF@5@5@+=feaafiart1ev1aaatCvAUfKttLearuWrP9MDH5MBPbIqV92AaeXatLxBI9gBaebbnrfifHhDYfgasaacH8akY=wiFfYdH8Gipec8Eeeu0xXdbba9frFj0=OqFfea0dXdd9vqai=hGuQ8kuc9pgc9s8qqaq=dirpe0xb9q8qiLsFr0=vr0=vr0dc8meaabaqaciGacaGaaeqabaqabeGadaaakeaacqWGdbWqdaaeqbqaaiabe67a4naaBaaaleaacqWGPbqAaeqaaaqaaiabdMgaPbqab0GaeyyeIuoakiabg2da9iabdoeadnaaqafabaGaeqOVdG3aaSbaaSqaaiabdMgaPbqabaaabaGaei4EaSNaemyAaKMaeiOoaOJaemyEaK3aaSbaaWqaaiabdMgaPbqabaWccqGH9aqpcqaIXaqmcqGG9bqFaeqaniabggHiLdGccqGHRaWkcqWGdbWqcqWGkbGsdaaeqbqaaiabe67a4naaBaaaleaacqWGPbqAaeqaaaqaaiabcUha7jabdMgaPjabcQda6iabdMha5jabg2da9iabgkHiTiabigdaXiabc2ha9bqab0GaeyyeIuoaaaa@5885@. The heavier weight *CJ *imposed on the errors originating from the negative points enforces a low false positive rate for unbalanced training sets [39].

The final prediction system was constructed as follows. The entire set of proteins with single-localization was used as a training set; and the optimal value for each parameter of the SVMs for the training set was taken as the average value of the optimal parameters obtained from the LOOCV procedure. Using these optimized parameters, final binary classifies were learned from the training set. The evaluation for the set of multi-localization proteins was based on this final prediction system. The framework for the overall training and testing procedures is illustrated in Figure S1 in the supplementary file [see Additional file 2].

## Availability and requirements

Project name: Subnuclear Compartments Prediction System (Version 1.0)

Project home page: 

Operating system(s): Linux

Programming language: Perl

License: None

Any restrictions to use by non-academics: None

## Authors' contributions

ZL designed the methodology and developed the programs. YD contributed with ideas on overall design, implementation, and assisted with drafting the manuscript.

## Supplementary Material

Additional File 1 This file includes Table S1 – Prediction for multi-localization proteins. A correct prediction is counted if one of the localizations is predicted. Click here for file

Additional File 2 This file includes Figure S1 – Diagrammatic view of our SVM-based system for the prediction of protein subnuclear localizations. Click here for file
