# Supplementary material for: An SVM-based system for predicting protein subnuclear localizations
Source: BMC Bioinformatics. 2005 Dec 7;6:291. doi: 10.1186/1471-2105-6-291 (PMC1325059; doi:10.1186/1471-2105-6-291)
Supplement: Additional File 1 — This file includes Table S1 – Prediction for multi-localization proteins. A correct prediction is counted if one of the localizations is predicted. [file 1471-2105-6-291-S1.doc]

Supplementary materials- Additional file1

## Table S1 - Prediction for multiple-localization proteins. A correct prediction is counted if one of the localizations is predicted.

| **SwissProt ID or Entrez Protein ID** | **Localizations Reported** | **Localization Predicted** | **Accumulated Accuracy** |
| --- | --- | --- | --- |
| O15350 | 5 1 | 1 | 1/1 |
| O60563 | 3 1 | 1 | 2/2 |
| Q9UKM6 | 4 5 | 6 | 2/3 |
| Q9UHQ1 | 2 5 | 6 | 2/4 |
| P05455 | 6 5 | 6 | 3/5 |
| Q12906 | 4 5 6 | 6 | 4/6 |
| Q00597 | 4 5 | 2 | 4/7 |
| NP_002844 | 5 1 | 1 | 5/8 |
| O75618 | 6 5 | 6 | 6/9 |
| P14678 | 3 6 | 3 | 7/10 |
| Q01860 | 3 6 | 6 | 8/11 |
| Q12905 | 4 6 | 6 | 9/12 |
| P07910 | 3 6 | 6 | 10/13 |
| Q14331 | 3 6 | 6 | 11/14 |
| Q99J95 | 3 6 | 6 | 12/15 |
| P06748 | 4 6 | 6 | 13/16 |
| BAA24955 | 3 1 | 6 | 13/17 |
| Q15554 | 6 1 | 6 | 14/18 |
| O75714 | 5 1 6 4 | 6 | 15/19 |
| Q15596 | 5 1 | 5 | 16/20 |
| Q13185 | 6 1 | 6 | 17/21 |
| P23197 | 4 1 | 6 | 17/22 |
| P06730 | 3 1 | 2 | 17/23 |
| Q08211 | 5 1 6 | 6 | 18/24 |
| P38398 | 4 1 | 1 | 19/25 |
| Q13535 | 6 1 | 2 | 19/26 |
| O43254 | 6 1 | 2 | 19/27 |
| P54132 | 6 1 | 1 | 20/28 |
| Q16587 | 3 5 | 4 | 20/29 |
| P40337 | 6 5 | 5 | 21/30 |
| Q02880 | 6 5 | 6 | 22/31 |
| O14746 | 6 5 | 6 | 23/32 |
| Q02447 | 2 5 | 5 | 24/33 |
| AAD27754 | 4 5 | 2 | 24/34 |
| NP_006297 | 4 5 | 6 | 24/35 |
| Q13573 | 3 5 | 3 | 25/36 |
| NP_037367 | 6 5 | 3 | 25/37 |
| Q92841 | 6 5 | 6 | 26/38 |
| P24386 | 2 5 | 1 | 26/39 |
| P26599 | 6 5 | 6 | 27/40 |
| P17844 | 6 5 3 4 | 6 | 28/41 |
| Q15233 | 6 5 | 3 | 28/42 |
| O15381 | 6 5 | 6 | 29/43 |
| O75694 | 2 5 | 2 | 30/44 |
| Q9H6W3 | 6 5 | 6 | 31/45 |
| Q9ESU6 | 4 5 | 3 | 31/46 |
| G01161 | 4 5 | 4 | 32/47 |
| Q13263 | 4 5 | 1 | 32/48 |
| O95163 | 6 5 | 6 | 33/49 |
| Q16665 | 6 5 | 1 | 33/50 |
| Q13547 | 4 5 | 6 | 33/51 |
| Q9ULX6 | 4 5 | 6 | 33/52 |
| P22087 | 4 5 6 | 6 | 34/53 |
| Q9NPI8 | 4 5 | 2 | 34/54 |
| Q03468 | 6 5 | 6 | 35/55 |
| Q9UBC3 | 4 5 | 6 | 35/56 |
| P78527 | 6 5 | 2 | 35/57 |
| Q99741 | 4 5 | 6 | 35/58 |
| P53564 | 2 5 | 5 | 36/59 |
| P49716 | 4 5 | 3 | 36/60 |
| P17676 | 4 5 | 3 | 36/61 |
| P09038 | 4 5 6 | 6 | 37/62 |
| NP_524576 | 2 5 | 1 | 37/63 |
| O15360 | 4 5 | 2 | 37/64 |
| P26368 | 3 6 | 6 | 38/65 |
| NP_473357 | 3 6 | 3 | 39/66 |
| O75937 | 3 6 | 6 | 40/67 |
| Q07955 | 3 6 4 | 3 | 41/68 |
| P70281 | 4 6 | 6 | 42/69 |
| Q01130 | 3 6 4 | 3 | 43/70 |
| P12750 | 3 6 | 6 | 44/71 |
| Q9BQ39 | 3 6 | 6 | 45/72 |
| O43143 | 3 6 | 6 | 46/73 |
| P55769 | 3 6 | 6 | 47/74 |
| P13010 | 4 6 | 6 | 48/75 |
| P12956 | 2 6 4 | 6 | 49/76 |
| O00532 | 4 6 | 6 | 50/77 |
| P04792 | 3 6 | 3 | 51/78 |
| P38159 | 3 6 | 3 | 52/79 |
| Q15365 | 3 6 | 6 | 53/80 |
| Q14103 | 3 6 | 6 | 54/81 |
| P22626 | 3 6 | 6 | 55/82 |
| P09651 | 3 6 | 6 | 56/83 |
| Q9NZM1 | 2 6 | 6 | 57/84 |
| Q9Y6D6 | 2 6 | 1 | 57/85 |
| O75934 | 3 6 | 2 | 57/86 |
| Q16629 | 3 6 | 3 | 58/87 |
| Q93068 | 6 1 2 | 5 | 58/88 |
| P06454 | 5 1 | 6 | 58/89 |
| P08578 | 3 6 | 6 | 59/90 |
| P13641 | 3 6 | 6 | 60/91 |
| NP_003851 | 4 2 | 6 | 60/92 |
| Multiple-localization Overall Accuracy | | | 65.2% |
