# Supplementary material for: An SVM-based system for predicting protein subnuclear localizations
Source: BMC Bioinformatics. 2005 Dec 7;6:291. doi: 10.1186/1471-2105-6-291 (PMC1325059; doi:10.1186/1471-2105-6-291)
Supplement: Additional File 2 — This file includes Figure S1 – Diagrammatic view of our SVM-based system for the prediction of protein subnuclear localizations. [file 1471-2105-6-291-S2.doc]

Supplementary materials- Additional file2.

**Figure S1 - Diagrammatic view of our SVM-based system for the prediction of protein subnuclear localizations.**

**5-fold CV**

Evaluate

Predictions

Raw Data

from NPD

Protein Label

P49959 1

Q9P0I1 2

Q13627 3

… …

LOO-CV test sequence

Multiple

Localizations

Test Set

Single

Localization

Dataset

Parameter Tuning

Training

Set

Validation

Set

SVM Models

Evaluation Models

Final Models

Sequences Submitted from Internet

LOO-CV Evaluation Result

Prediction

Results

**LOO-CV**

Prediction
